# Protein function prediction by massive integration of evolutionary analyses and multiple data sources

**DOI:** 10.1186/1471-2105-14-S3-S1

**Published:** 2013-02-28

**Authors:** Domenico Cozzetto, Daniel WA Buchan, Kevin Bryson, David T Jones

**Affiliations:** 1 Bioinformatics Group, Department of Computer Science, University College London, Gower Street, London, WC1E 6BT, UK

## Abstract

**Background:**

Accurate protein function annotation is a severe bottleneck when utilizing the deluge of high-throughput, next generation sequencing data. Keeping database annotations up-to-date has become a major scientific challenge that requires the development of reliable automatic predictors of protein function. The CAFA experiment provided a unique opportunity to undertake comprehensive 'blind testing' of many diverse approaches for automated function prediction. We report on the methodology we used for this challenge and on the lessons we learnt.

**Methods:**

Our method integrates into a single framework a wide variety of biological information sources, encompassing sequence, gene expression and protein-protein interaction data, as well as annotations in UniProt entries. The methodology transfers functional categories based on the results from complementary homology-based and feature-based analyses. We generated the final molecular function and biological process assignments by combining the initial predictions in a probabilistic manner, which takes into account the Gene Ontology hierarchical structure.

**Results:**

We propose a novel scoring function called COmbined Graph-Information Content similarity (COGIC) score for the comparison of predicted functional categories and benchmark data. We demonstrate that our integrative approach provides increased scope and accuracy over both the component methods and the naïve predictors. In line with previous studies, we find that molecular function predictions are more accurate than biological process assignments.

**Conclusions:**

Overall, the results indicate that there is considerable room for improvement in the field. It still remains for the community to invest a great deal of effort to make automated function prediction a useful and routine component in the toolbox of life scientists. As already witnessed in other areas, community-wide blind testing experiments will be pivotal in establishing standards for the evaluation of prediction accuracy, in fostering advancements and new ideas, and ultimately in recording progress.

## Background

Deriving useful knowledge from genomic data depends critically on the ability to accurately assign biological roles to genes and their products. The only conclusive way of characterizing protein function is by experimental determination, although interpretation may still lead to errors. Even with high-throughput screenings, functional assays remain time consuming, expensive and technically challenging. Additionally, experimental techniques target known aspects of function, which may provide a one-sided picture of otherwise multi-faceted concepts.

Currently, the 'gold standard' for high-quality database annotation consists of manual curation by experts based on literature searching and bioinformatics analyses [1,2]. However, this route is also time consuming and the number of sequences manually annotated clearly lags behind the exponential growth in the number of known sequences [3]. Consequently, most entries in UniProt [4] include electronically inferred annotations, often of unknown reliability. Therefore, the development of reliable automatic function prediction tools is a major goal, since it is the only way of keeping database annotations updated as more and more sequence data pours in.

Simple function predictors implement annotation transfer from well characterized proteins with high sequence similarity to the target. However, they achieve rather limited performance [5], principally due to the challenges posed by multi-domain proteins, and by paralogous and analogous genetic relationships. More sophisticated methods aim at detecting local signatures indicative of function such as sequence motifs, domains, or domain architectures [6,7]. Previous work has also benefitted from considering phylogenetic analyses [8], the hierarchical structure of GO [9] and the co-occurrence of its terms in high-quality database annotations [10].

Neural networks [11] and support vector machines [12] have been successfully trained to recognize sequence-derived patterns indicative of generic functional roles. The requirement of sufficient training examples for robust modelling often restricts such methods to making broad functional assignments. While such predictions do not help design specific experiments, they can be valuable for proteins with distant or no detectable sequence relatives. Other homology-free methods assume that functionally associated genes are co-expressed and their products interact with each other in some capacity. Usually these supervised or unsupervised approaches exploit high-throughput gene expression or protein-protein interaction data largely to assign proteins to known biological processes [13-15].

Additionally, large-scale screening data have also become available, and genomic data integration is a promising avenue with the potential to overcome the intrinsic limitations of techniques utilizing individual information sources. Integrative strategies can provide both increased coverage of the protein universe and more confident functional assignments when supporting evidence can be gathered from different studies [16]. A wide array of machine learning and computational statistics tools have been applied to effectively combine heterogeneous data types, including those mentioned, as well as information about gene co-localization, transcription factor binding, phenotype annotations, disease associations and biomedical literature. Interested readers are referred to [17] and citations therein.

As in other research areas of computational biology, the need to critically test and compare so many approaches has recently prompted the establishment of community-wide experiments. The MouseFunc experiment blindly tested independent integrative methods by providing common *M. musculus *datasets with limited homology information for both training and internal benchmarking purposes [18]. The CAFA challenge provided a more realistic setting to comprehensively undertake blind testing of generic function prediction methods. The key task was to predict Gene Ontology (GO) [19] terms for 48,298 target proteins from a diverse range of species including seven eukaryotes and eleven prokaryotes. Prior to the experiment start, UniProtKB/Swiss-Prot curators had reviewed most of the corresponding entries, but approximately 30% had no GO terms for the Molecular Function (MF) or Biological Process (BP) categories. The remaining targets were principally annotated with electronically inferred annotations which were rarely specific (i.e. terms associated with nodes at the lowest level of the GO hierarchy).

Here we first detail the integrative approach we used for this annotation challenge, which combines into a single framework a wide variety of tools and biological information sources encompassing sequence, gene expression and protein-protein interaction data. We then benchmark the overall methodology and its components using a novel scoring scheme to measure the information overlap between predicted and reference GO terms. Finally, we discuss the lessons we learnt from the first round of CAFA.

## Methods

Our approach to predicting function is based around combining a broad range of large scale function annotation methods and data sources. Unless otherwise noted, each component method was calibrated against a benchmark set, with the estimated precision used to determine the "confidence" of each method. The performance of all methods was independently evaluated on 595 CAFA targets that received experimental functional annotations between January and July 2011.

### Benchmarking and estimation of prediction accuracy

A set of 1,546 well characterized proteins from UniProtKB/Swiss-Prot was selected for internal benchmarking and calibration purposes. We defined the set of reference functional annotations as the reported GO terms for the Molecular Function (MF) and Biological Process (BP) categories along with all those more general descriptions linked by "is a" (parental) relationships in the ontology. Each method described below was then run independently on this benchmark set and the predicted GO terms were collated. We then independently binned the predictions into equally sized groups based on their raw scores - e.g. bit scores from PSI-BLAST [20] or raw SVM scores from FFPRED [12] - and calculated the number of true and false positives - TP and FP respectively - for each group. From these statistics we derived precision values ($Prec=\frac{TP}{TP+FP}$) that we subsequently fitted to the raw scores, assuming a standard logistic function ($P_{t}=\frac{a}{1+be^{-ct}}$). We used these regression models (one model per method) to estimate the precision of each prediction given a particular raw score.

With the above benchmark in hand, a range of different component methods were evaluated. We preselected only methods which had significantly better than random performance on the benchmark set, and the final component methods are as follows.

### GO term prediction using PSI-BLAST

The most basic method employed in this work was a standard PSI-BLAST search against the UniRef90 [21] database, using 3 iterations, and an E-value threshold of 1x10^-3 ^for both hit selection and profile inclusion. Then, for each target sequence we report the MF and BP GO terms associated with any matches with alignment coverage of at least 85% of its length. In order to estimate the accuracy of the predictions, we selected the highest bit score of the alignments between the target and those sequences annotated with that term and converted this bit score into a value in [0.00, 1.00] given the logistic regression model obtained during the benchmark.

### GO term prediction from UniProtKB/Swiss-Prot text-mining

For targets which already had descriptive text, keywords or comments in UniProtKB/Swiss-Prot, GO terms were assigned using a naïve Bayes text-mining approach [22]. To start with, the relative frequencies of occurrence of single words and consecutive pairs of words were computed for all records where a GO term appears (f(word|GO)), along with the equivalent frequency in records where the GO term is absent (f(word|~GO)) in the data bank. To avoid zero counts, a pseudocount of 1 was used for all probability estimates. From this raw data, the conditional probability of each GO term based on the occurrence of each word group was estimated as follows:

$$p\left(GO|word\right)\approx \frac{f\left(word|GO\right)}{f\left(word\text{|}GO\right)+f\left(word|\sim GO\right)}$$

By combining these probabilities (by converting to log likelihood scores and summing), naive Bayes classification was carried out on the raw text from a particular UniProtKB/Swiss-Prot entry and likely GO terms predicted.

To further extend the scope of the textual analysis, words occurring in the different UniProtKB/Swiss-Prot record types were recorded, and some simple pre-parsing of feature (FT) records was also carried out. Specifically, in cases where the FT records specified residue numbers or residue number ranges, these residue numbers were placed into bins of width 50 and converted into word tokens (NUMVAL0...NUMVALn). This allowed a simple form of feature-based function prediction to be carried out. For example, the following UniProtKB/Swiss-Prot FT record:

TOPO_DOM 25 308 Extracellular (Potential),

would be parsed into the following five tokens:

FT:TOPO_DOM FT:NUMVAL0 FT:NUMVAL6 FT:Extracellular FT:Potential.

These tokens are treated in exactly the same way as words found in other record types.

### GO term prediction from amino acid trigram mining

The same naïve Bayes classification approach that was used to relate GO terms to descriptive statements in the UniProtKB/Swiss-Prot records was also applied to the amino acid sequences. In this case, rather than words in description lines, trigrams of amino acids were counted across the whole sequence database. From this raw data, the conditional probability of each GO term based on the occurrence of each trigram was estimated in the form of log likelihood scores. For all trigrams in the target sequence, log likelihood scores for particular GO terms were calculated by summing the individual log likelihood scores for all referenced GO terms in the training data.

### GO term prediction from sequence features (FFPRED)

FFPRED [12] was also used to make GO term predictions for eukaryotic targets. FFPRED starts by predicting a diverse range of sequence features, which include secondary structure elements, disordered regions, signal peptides, glycosylation sites, and several others. These features are then analysed by a series of Support Vector Machines (SVMs) to assign GO terms from a subset of 197.

### GO term prediction from orthologous groups

In addition to pairwise close homologues, more distant evolutionary relationships were obtained from the eggNOG (v2.0) collection of orthologous groups [23]. For each target that could be assigned to an orthologous group, all GO terms found in GOA [24] for all members of the same group were assigned with an estimated precision being calculated as the fraction of group members that shared the given GO term.

### GO term prediction from profile-profile comparison

In addition to pairwise close homologues, very distant evolutionary relationships were obtained from the use of profile-profile comparisons. PSI-BLAST was used to compute Position Specific Scoring Matrices (PSSMs) for each entry in UniProtKB/Swiss-Prot along with every target sequence. Each target PSSM was then compared against the set of UniProtKB/Swiss-Prot PSSMs according to the following scoring scheme based on the dot products of the two PSSM vectors X (from the target sequence PSSM) and Y (from the UniProtKB/Swiss-Prot entry PSSM):

$$S\left(X,Y\right)=\frac{\sum_{i=1}^{20}X_{i}^{TF}Y_{i}}{\sum_{i=1}^{20}X_{i}^{TF}}+\frac{\sum_{i=1}^{20}Y_{i}^{TF}X_{i}}{\sum_{i=1}^{20}Y_{i}^{TF}}$$

where *X^TF ^*and *Y^TF ^*are the respective target frequencies. A simple affine gap penalty was used with an opening penalty of 11 and an extension penalty of 1.

To predict GO terms, the profile-profile alignments were evaluated using a neural network with 4 input units, 4 hidden units and a single output unit. The 4 inputs were the profile-profile alignment score, the percentage sequence identity, the percentage alignment coverage of sequence 1 and the percentage alignment coverage of sequence 2. The single output represented the presence or absence of common GO terms between the two proteins. The neural network was then trained using the benchmark GO data set to distinguish alignments sharing common GO terms from those that do not. Training vectors were generated per GO term rather than per alignment, such that a pair of profiles having 5 common GO terms and say 3 mismatching GO terms would result in 8 separate training vectors (5 positive cases and 3 negative cases). This ensured that the posterior probabilities extracted from the trained neural network would more accurately represent probabilities of GO term occurrence. After training, the neural network output was calibrated to produce precision estimates using the procedure described above. Rather than using a separate data set, the same benchmark set used to train the network was used to calibrate the output. This was acceptable here because the eventual evaluation of the method would be carried out on "blinded" data not available at the time the neural network training took place.

### GO term prediction from high-throughput data sources (FunctionSpace)

For human proteins (and any closely related eukaryotic homologues) medium-to-low-confidence predictions of protein function were generated by an SVM regression method called FunctionSpace [25]. Human proteins are assigned coordinates in an 11-dimensional feature space and GO terms assigned from annotated close neighbours in this space. The 11 dimensions represent pairwise sequence similarity, predicted cellular localization, secondary structure similarity, transmembrane topology, disordered segment features, sequence-derived domain architecture, structure-based domain architecture, sequence domain fusion patterns, structural domain fusion patterns, protein-protein interactions and microarray data. The microarray features were derived from bi-clustering of 81 publicly available experimental microarray datasets in order to identify which experiments and genes had maximal predictive value for specific GO categories. In total, over 49,231 features were combined to train the 11 SVM regression models and their outputs are integrated using a further second layer regression SVM.

### Integration of methods and post-processing

To produce a final consensus set of predictions, separate prediction files were generated for each of the component methods described above, and these predictions were combined using a network propagation algorithm based on the GO graph structure. We start by defining $D_{ij}$ as the difference in depth between two GO terms *i *and *j*. For this to be defined, a direct path between the two terms must exist that does not include the root node. For pairs of terms that have no direct connecting path, $D_{ij}$ is left undefined, and in programming terms is given a NULL value to flag this situation. Where the sign of $D_{ij}$ is positive, this indicates that term *i *is deeper (more specific) than term *j *and vice versa for negative values.

As a first step, all predicted terms from each component method are collated into a single set of terms. This set is not extended further e.g. by adding higher level or intermediate terms. For cases where *n *methods predict the same term, the combined precision for the common term (P') is estimated as follows:

$$P'=1-\prod_{i=1}^{n}\left(1-\alpha P_{i}\right)$$

where α∈[0,1] is a constant attenuation parameter (set to 0.9 in this case) and *P_i _*the estimated precision for prediction *i*. The use of this attenuation parameter is to prevent the final estimated precision values from reaching a maximum of 1.0 due to a single 'vote' from just one of the methods.

For each target, all lower level term scores were propagated up to higher level terms on the same path to root according to the following procedure:

FOREACH GO term *i*

   FOREACH GO term *j≠i*

      IF *D_ij _≠ NULL *AND *D_ij _< 0 *THEN

         $P_{i}^{'}\leftarrow 1-\left(1-P_{i}\right)\left(1-P_{j}\right)$

   END IF

The final list of terms was produced by first ranking the terms according to the final chained precision estimates calculated as described above. To exclude mutually incompatible term pairs from the final list, each pair of predicted GO terms was checked for co-occurrence in UniprotKB. For any pairs of terms for which *D_ij _≠ NULL *and which were not seen to co-occur in UniProtKB, the term with lowest estimated precision was deleted from the list. Where a pair of such terms had equal precision values, both were kept.

### COGIC scores

In the light of the reference experimental data released by the assessors, we analysed our own predictions using a novel scoring scheme that takes into account each term specificity and confidence value. We propose to calculate different Graph-Information Content (simGIC) similarity scores [26] for overlapping subsets of GO term predictions compared to their reference annotations, and to combine them such that the final measure lies in [0.0, 1.0].

$$simGIC\left(A,B\right)=\frac{\sum_{t\in A\cap B}IC\left(t\right)}{\sum_{t\in A\cup B}IC\left(t\right)}$$

where *IC(t) *is the Information Content of *t *- an empirical measure of its specificity. For each term *t*, we first counted its occurrence *n(t) *in UniProtKB/Swiss-Prot functional assignments. We chose to use only experimentally supported evidence codes; this reduces the size of the reference corpus, but also helps minimize assessment bias towards homology-based annotations [27]. The initial counts were then propagated to the ancestral nodes and IC values computed as $IC\left(t\right)=-log\frac{n\left(t\right)}{n\left(r\right)}$ where *r *is the root of the ontology.

For each target, four overlapping subsets of predicted GO terms are calculated based on confidence scores ≥ 0.75, 0.5, 0.25 and 0, and denoted as *P_1_, P_2_, P_3 _*and *P_4 _*respectively. These sets as well as the set *R *of reference GO terms are expanded by adding all ancestral nodes. The simGIC scores *S*_1_, *S*_2_, *S*_3_, and *S*_4 _are then calculated by comparing *R *with *P*_1_, *P*_2_, *P*_3 _and *P*_4 _respectively and the final COmbined simGIC (COGIC) score for a given protein target is given by

$$COGIC\left(P,R\right)=\frac{S_{1}+S_{2}+S_{3}+S_{4}}{4},$$

thus ensuring that correct predictions with higher confidence scores are rewarded more. The COGIC score equals 1 when $R=P_{1}=P_{2}=P_{3}=P_{4}$, i.e. all predicted GO terms are validated and their confidence scores are greater than or equal to 0.75. Its value is 0 if $R\cap P_{1}=R\cap P_{2}=R\cap P_{3}=R\cap P_{4}=\left\{r\right\}$, where *r *is the root of the ontology (that is the root node is the only common element between the predicted and reference annotations).

### Baseline predictions of GO terms

We compare the performance of our integrative approach with two independent naïve predictors that the assessors had run against the same test set. The Priors algorithm determines the most frequent 10,000 GO terms in UniprotKB/Swiss-Prot and assigns them to each target using their frequencies as confidence values. The BLAST method transfers GO terms from the hits of a BLAST [28] search against a database of experimentally characterized proteins with default parameters; the confidence values is the scaled sequence identity between the target and the most similar hit bringing the annotation.

## Results

### Role of the component methods

The organizers initially released 48,298 target proteins at the beginning of the experiment. At the Automated Function Prediction meeting in July 2011, method performance was benchmarked against 595 proteins that had been experimentally characterized in the intervening months. This set comprises of 366 proteins with annotated Molecular Function terms (MF subset), and 436 proteins with Biological Process terms (BP subset). We use these data provided by the organizers as a blind test set for the analyses below.

Table 1 shows the extent to which the component methods contributed to the submissions of the team Jones-UCL, as well as to the data officially evaluated by the assessors. Profile-based and text mining approaches were able to assign functional classes to most targets, but only in combination could the entire test set be completely covered. As expected, FunctionSpace produced GO term assignments for lower numbers of targets; as it had been originally designed for annotation of human proteins, it could only be applied to eukaryotic sequences. Unfortunately, the extremely limited overlap between predicted and assessed targets prevents us from drawing any general and sound conclusions about this method's contribution and performance. The corresponding data are therefore omitted from the following analyses and discussion.

**Table 1:** Coverage statistics of the CAFA test set.

Method	Coverage of Targets			
	Released	Assessed	MF	BP
Orthologous Groups	49.15%	60.84%	61.75%	61.01%

PSI-BLAST	83.83%	90.42%	89.89%	89.45%

Profile-Profile Comparison	99.87%	99.50%	99.73%	99.31%

Amino Acid Trigram Mining	49.64%	47.73%	50.00%	47.25%

Swiss-Prot Text Mining	89.19%	94.96%	95.08%	94.50%

FFPRED	72.40%	56.30%	50.82%	56.65%

FunctionSpace	11.27%	7.56%	6.83%	8.03%

**Jones-UCL**	**100.00%**	**100.00%**	**100.00%**	**100.00%**

For each method, the columns report the percent of targets that were assigned GO terms relative to the entire benchmark (Released), the subset officially evaluated (Assessed), the evaluated molecular function predictions (MF), and the assessed biological process assignments (BP).

**Figure 1:**
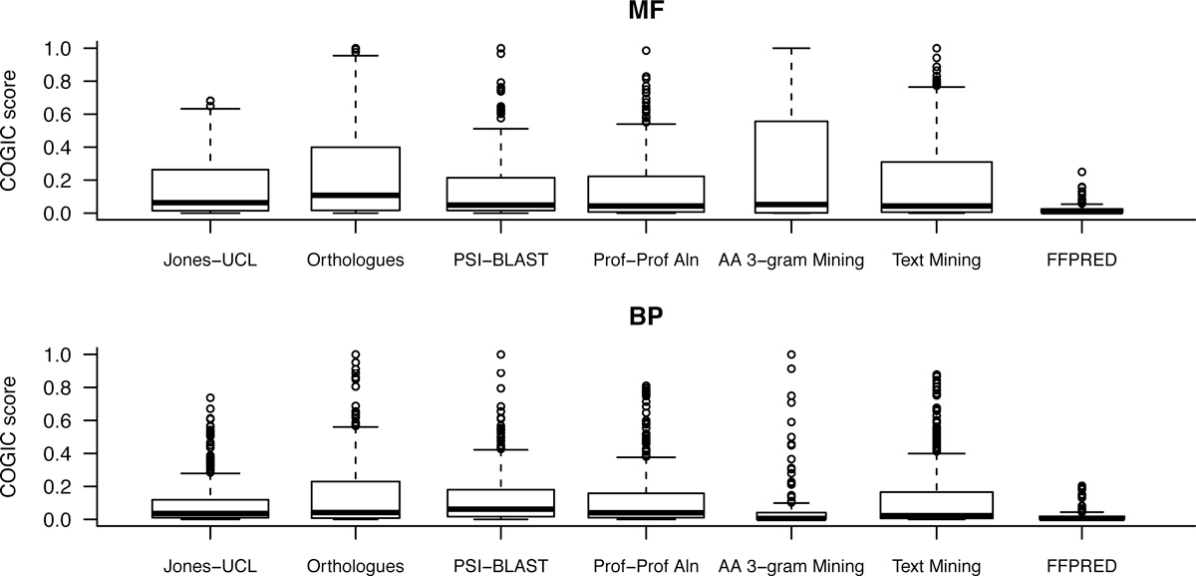
**Performance of Jones-UCL in comparison with the component methods**. Comparison of the COGIC score distributions achieved by the individual predictors on the MF (upper panel) and BP (lower panel) benchmark sets. Each box spans from the first to the third quartile of the corresponding distribution; the median value is highlighted as a thick line, while putative outliers are plotted as empty circles. The width of each box is proportional to the number of targets predicted and officially assessed. Some names have been shortened ("Orthologues" for Orthologous Groups, "Prof-Prof Aln" for Profile-Profile Alignment, "AA 3-gram Mining" for amino acid trigram mining and "Text Mining" for Swiss-Prot Text Mining).

We performed target-by-target numerical evaluation of the remaining predictions using the COGIC similarity score and plotted the results in Figure 1. The performance of homology-based methods displays an expected pattern both within and across the MF and BP data sets. Orthologous Groups generally scores higher than PSI-BLAST and Profile-Profile Alignment, which in contrast provide higher coverage. In line with previous findings, the shift of the score distributions towards the top end is larger for molecular function predictions rather than the biological process ones [5,26]. The amino acid trigram mining classifier appears very competitive with the other homology-based approaches on the MF benchmark. Yet, the picture does not take into account the data in Table 1 and a more detailed comparison (see additional file 1) would suggest that amino acid trigram mining only made predictions for "easy" targets in MF. The scores for FFPRED are unsurprisingly low: this machine-learning tool had been trained to predict a restricted set of general functional classes specifically for human proteins with distant or no detectable sequence relatives.

In order to investigate the usefulness of each component method, we initially tried to measure their exclusive scope with regard to the numbers of targets and GO terms officially evaluated. Unfortunately, we couldn't achieve conclusive results due to the large overlap among the sets of predicted targets (only 5 proteins were annotated by a single method). Furthermore, no method output GO term assignments that (i) were not explicitly predicted or implied by the annotations made by other components, and that (ii) were reference annotations used for assessment or one of their ancestors. Consequently, we were unable to identify any single component method which was pivotal for the success of the overall strategy.

### Overall performance of the team Jones-UCL

**Figure 2:**
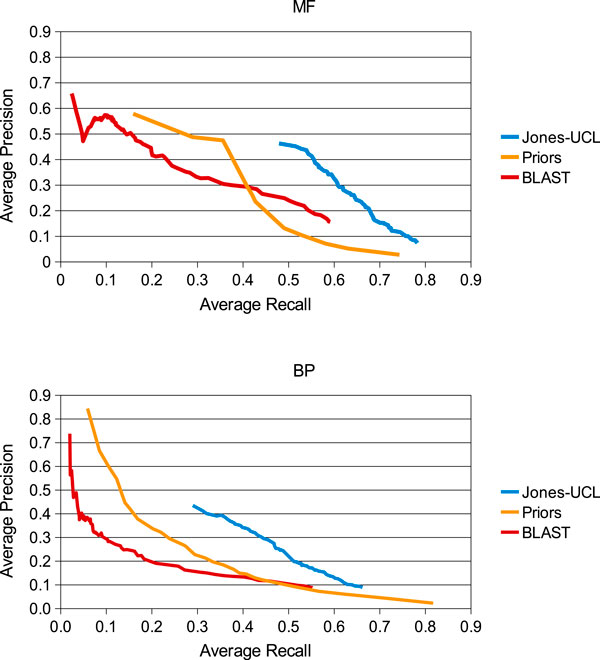
**Average precision against average recall for the Jones-UCL and the baseline methods**. The graph shows the performance of our aggregate method in comparison with the simple annotation strategies calculated by the CAFA assessors Priors and BLAST. Each data point represents the average precision and recall across the entire set of 595 targets with functional annotations as of July 2011.

Figure 2 shows the performance of the final integrative strategy with attenuation parameter α = 0.9 in comparison to that of the naïve predictors from the assessors. In this case, we plot the average precision and recall values across the whole set of 595 targets as provided in the official assessment. Our approach clearly outperforms the baseline methods for both molecular function and biological process predictions beyond a recall of 0.5 and 0.3 respectively. After the official assessment data were released, we investigated how the method would perform as the attenuation parameter α varies. While the resulting statistics and plots did not exhibit drastic changes, we note that decreasing α tends to generate predictions with lower average recall and higher average precision.

We calculated the distributions of COGIC scores for the group Jones-UCL and compared them with the other data in Figure 1. The final integrative and post-processing steps affect the GO term lists and their confidence scores in interesting ways. On the one hand, Jones-UCL does not consistently improve over all components - e.g. orthologues and text mining tend to achieve slightly higher prediction accuracy scores. On the other hand, it records a COGIC score of 0 for fewer targets than the component methods. Overall, no single component yielded significantly more accurate predictions than the aggregate method.

**Figure 3:**
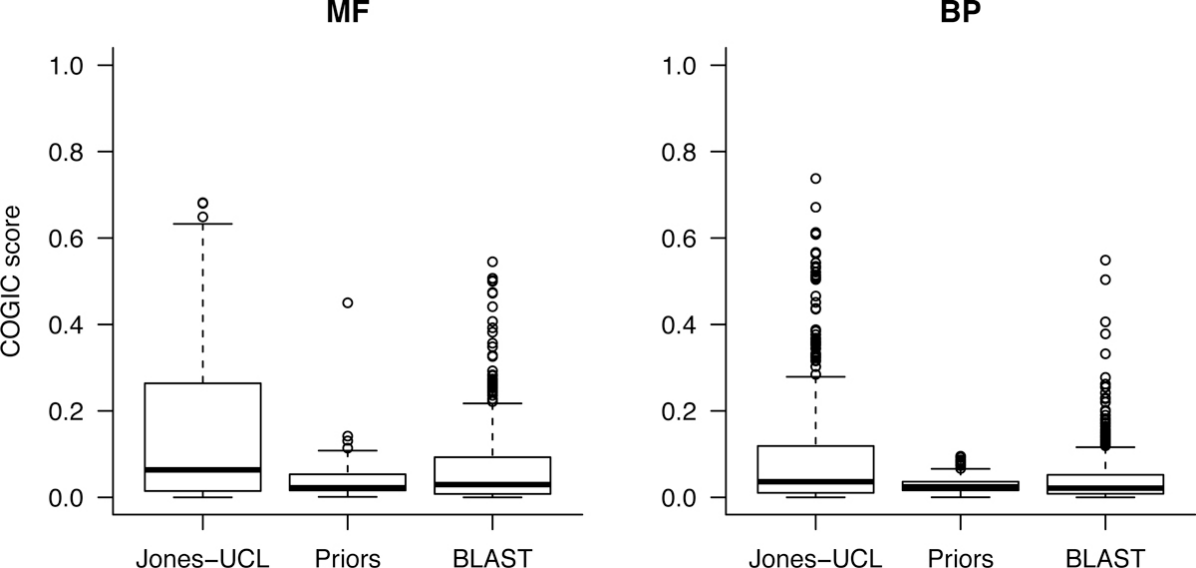
**Performance comparison based on COGIC scores**. The boxplot recapitulates the target-based distributions for the Jones-UCL prediction team as well as for the naïve algorithms Priors and BLAST.

Next, we compared the performance of our aggregate method with the two baseline predictors Priors and BLAST. The plots in Figure 3 clearly show that Jones-UCL scores are higher than the others on both ontologies. We also tested the null hypothesis that the performance of Jones-UCL is not statistically different from that of the naïve approaches using two-sided pair-wise Wilcoxon rank sum tests and Table 2 gives the resulting p-values. At a p-value cut-off of 10^-5^, our approach significantly outperforms both BLAST and Priors in assigning molecular function and biological process terms.

**Table 2:** Statistical comparisons of prediction performance.

Method	Method	Set	n	p-value
Jones-UCL	Priors	MF	366	7.75E-06

Jones-UCL	BLAST	MF	351	3.37E-23

Jones-UCL	Priors	BP	436	3.43E-15

Jones-UCL	BLAST	BP	387	6.62E-12

The performance of pairs of methods was compared separately on the two benchmark subsets MF and BP. We tested the null hypothesis that the distributions of the COGIC scores over the set of common targets were indistinguishable with a paired two-sided Wilcoxon sum rank test. The table reports the number n of common targets and the p-value.

**Figure 4:**
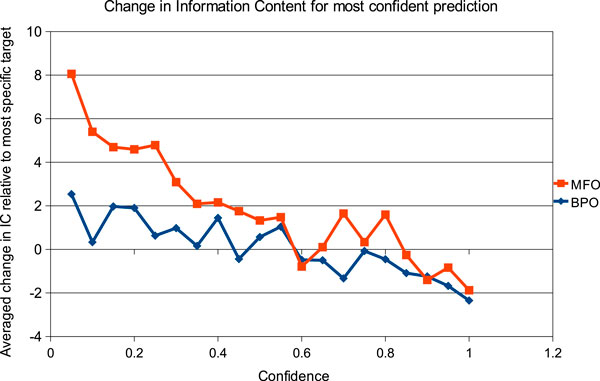
**Relationship between confidence scores and GO term specificity**. Positive values indicate that on average the assignments made by the team Jones-UCL at a given confidence level were more specific than the corresponding experimental annotations. Negative values indicate the converse. Molecular Function (MF) and Biological Process (BP) data were analysed and plotted separately.

An inverse relationship exists between the specificity of the predicted GO terms and their confidence scores, as a result of the back-propagation algorithm used. However, it is not immediately clear whether our method tends to be more or less specific than the target annotations. To investigate this, we analysed how the difference in specificity between predicted and reference GO terms varies as a function of the predicted confidence scores. To this end, we considered pairs (x,y) of predicted and experimental GO terms where (i) *x *and *y *lie on the same path to root, and (ii) there are no descendants of *x *predicted for the same target. For such pairs, we calculated $IC\left(x\right)-IC\left(y\right)$ and in Figure 4 we plot the average of these values across all targets against the confidence scores assigned to *x*. The plot shows the expected inverse correlation between our confidence scores and the average semantic distance from experimental annotations.

At lower confidence values, the team Jones-UCL assigned more specific GO terms than those found in UniprotKB/Swiss-Prot annotations. Further studies and additional experimental data will be necessary to determine whether these predictions should be regarded as false positives or not. Conversely, the most confident predictions typically consist of more general terms than those experimentally validated. This trend stems essentially from homology-based annotation transfers: specific functional annotations can only be made between very close homologues. As evolutionary distance increases, more general terms can be assigned confidently, because distinguishing between paralogues and orthologues becomes harder and harder due to limited data available.

## Discussion

This study confirms that combining the strengths of different approaches provides increased coverage of the protein sequence space and more accurate function predictions. To make our integrative strategy available to the community as a reliable and fast fully automated system, we need to address a few interrelated methodological questions. These relate to the identification of the most valuable component methods and to their effective combination.

The preliminary analysis presented here did not help estimate properly the individual contributions made by each component. In future studies, we propose to identify pivotal components by contrasting the performance of the current approach with its "leave-one-out" variants. More refined statistical analyses may also be required to account for the relatedness between separate components (e.g. the homology-based ones).

The final GO term predictions and their confidence scores were generated using heuristic methods. It will be interesting to explore alternatives that do not weight all the components evenly. This may be implemented by tailoring the attenuation parameters to the specific data sources and tools or - more flexibly - by training high-level classifiers such as SVMs or naïve Bayes approaches. Of course, this choice may affect the way we will be able to select the subset of components that maximizes prediction scope and accuracy.

The proposed COGIC score builds on previous efforts to numerically evaluate the semantic similarity between functional annotations. It represents an initial attempt to provide a normalized measure of information overlap between the predicted and benchmark annotations for a protein target, which explicitly accounts for prediction confidence values. The critical evaluation of protein function predictions is not a standard procedure yet, but it is drawing more and more attention. We hope that this work will contribute to further discussions and new ideas in the future.

## Conclusions

Methods for gene function predictions are continuously being devised and improved to account for the ever increasing size of public databases and the broad range of data sources at hand. Evaluating their scope and usefulness has always been difficult due to a lack of suitable test sets, evaluation metrics and additional experimental settings. The CAFA experiment has provided a unique opportunity and a real high-throughput use-case scenario where biologists need as much detailed, accurate and extensive functional annotations as possible.

Here we have detailed our integrative prediction approach entered at CAFA and have provided a complementary assessment of its performance. Our results are encouraging but overall highlight considerable room for improvement in the field. In line with previous findings the accuracy of molecular function predictions is higher than for biological process annotations. This applies to simple homology-based predictors and to more complex methodologies like the one described here, though as yet neither can be said to perform adequately.

Computational biologists evidently need to invest a great deal of effort to bring these methods up to an acceptable performance level. As already witnessed in other areas of bioinformatics, community-wide blind experiments will be pivotal in establishing standards for the evaluation of prediction accuracy and in fostering advancements and new ideas.

## Competing interests

The authors declare that they have no competing interests.

## Authors' contributions

DTJ conceived the study; all authors participated in its design and implementation; all authors drafted the manuscript, revised it critically and read and approved the final version.

## Supplementary Material

Additional file 1 **Boxplots of the COGIC score distributions on the benchmarks that were targeted by the amino acid trigram mining classifier**. figure with legend. Click here for file

## Declarations

This article has been published as part of *BMC Bioinformatics *Volume 14 Supplement 3, 2013: Proceedings of Automated Function Prediction SIG 2011 featuring the CAFA Challenge: Critical Assessment of Function Annotations. The full contents of the supplement are available online at URL. http://www.biomedcentral.com/bmcbioinformatics/supplements/14/S3.
