# Supplementary material for: Protein function prediction by massive integration of evolutionary analyses and multiple data sources
Source: BMC Bioinformatics. 2013 Feb 28;14(Suppl 3):S1. doi: 10.1186/1471-2105-14-S3-S1 (PMC3584902; doi:10.1186/1471-2105-14-S3-S1)
Supplement: Additional file 1 — Boxplots of the COGIC score distributions on the benchmarks that were targeted by the amino acid trigram mining classifier. figure with legend. [file 1471-2105-14-S3-S1-S1.pdf]

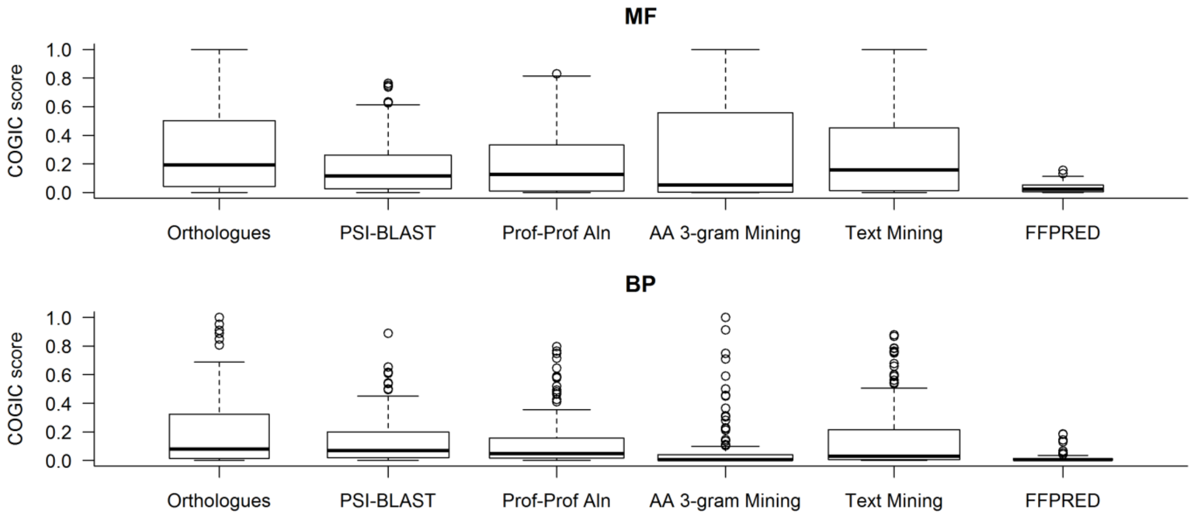

Additional figure 1: Supplementary comparisons of the COGIC score distributions. Individual predictors were contrasted on the subsets of the MF (upper panel) and BP (lower panel) benchmarks that were targeted by the Amino Acid Trigram Mining classifier. Each box spans from the first to the third quartile of the corresponding distribution; the median value is highlighted as a thick line, while putative outliers are plotted as empty circles. The width of each box is proportional to the coverage of the corresponding datasets. Groups are ordered as in Table 1 and some names shortened ("Orthologues" for Orthologous Groups, "Prof-Prof Aln" for Profile-Profile Alignment, "AA 3-gram Mining" for Amino Acid Trigram Mining and "Text Mining" for Swiss-Prot Text Mining).
